# MicroRNA 9 Is a Regulator of Endothelial to Mesenchymal Transition in Diabetic Retinopathy

**DOI:** 10.1167/iovs.64.7.13

**Published:** 2023-06-06

**Authors:** Eric Wang, Biao Feng, Subrata Chakrabarti

**Affiliations:** 1 Department of Pathology and Laboratory Medicine, Western University, London, Ontario, Canada

**Keywords:** diabetic retinopathy, endothelial-to-mesenchymal transition, microRNA, miR-9, epigenetic regulation

## Abstract

**Purpose:**

Diabetic retinopathy (DR) is a significant cause of blindness. Most research around DR focus on late-stage developments rather than early changes such as early endothelial dysfunction. Endothelial-to-mesenchymal transition (EndMT), an epigenetically regulated process whereby endothelial cells lose endothelial characteristics and adopt mesenchymal-like phenotypes, contributes to early endothelial changes in DR. The epigenetic regulator microRNA 9 (miR-9) is suppressed in the eyes during DR. MiR-9 plays a role in various diseases and regulates EndMT-related processes in other organs. We investigated the role miR-9 plays in glucose-induced EndMT in DR.

**Methods:**

We examined the effects of glucose on miR-9 and EndMT using human retinal endothelial cells (HRECs). We then used HRECs and an endothelial-specific miR-9 transgenic mouse line to investigate the effect of miR-9 on glucose-induced EndMT. Finally, we used HRECs to probe the mechanisms through which miR-9 may regulate EndMT.

**Results:**

We found that miR-9 inhibition was both necessary and sufficient for glucose-induced EndMT. Overexpression of miR-9 prevented glucose-induced EndMT, whereas suppressing miR-9 caused glucose-like EndMT changes. We also found that preventing EndMT with miR-9 overexpression improved retinal vascular leakage in DR. Finally, we showed that miR-9 regulates EndMT at an early stage by regulating EndMT-inducing signals such as proinflammatory and TGF-β pathways.

**Conclusions:**

We have shown that miR-9 is an important regulator of EndMT in DR, potentially making it a good target for RNA-based therapy in early DR.

Diabetes is a major global health issue, with its prevalence estimated to increase by nearly 50% in the next 25 years.^1^^,^^2^ Diabetes and its complications are major causes of mortality and quality of life reduction.^1^^,^^2^ Diabetic retinopathy (DR) remains the leading cause of blindness in working-aged adults.^3^^–^^7^ DR begins with asymptomatic hyperglycemic damage to the retinal microvasculature, eventually leading to functional changes in the eye that result in vision impairment and blindness.^7^^–^^12^ Current treatment approaches against DR target late-stage aspects of the disease and may not be effective in addressing early changes.^7^^,^^12^^,^^13^ Understanding the early molecular mechanisms behind the progression of DR is an important step to be able to tackle the problem at its root.

Vascular endothelial cells (ECs) support a wide variety of functions and help maintain homeostasis.^14^^–^^17^ ECs are constantly exposed to blood glucose and generally more susceptible to hyperglycemic damage.^18^ Hyperglycemic damage to ECs in the retinal vascular vasculature is at the root of DR and other diabetic complications.^19^^,^^20^ Hyperglycemia disrupts homeostatic regulation and leads to changes in ECs that result in endothelial dysfunction, which in turn paves the way for downstream changes in the tissues.^19^^,^^20^ One particular pattern of hyperglycemia-induced endothelial change is known as endothelial-to-mesenchymal transition (EndMT), where ECs transdifferentiate from a normal endothelial phenotype into a mesenchymal-like phenotype.^21^^–^^26^ EndMT is characterized by increased endothelial expression of extracellular matrix (ECM) proteins and mesenchymal proteins such as α-smooth muscle actin (α-SMA/ACTA2), transgelin (SM22/TAGLN), and fibroblast-specific protein (FSP/S100A4), accompanied by a reduction in the expressions of classical endothelial proteins such as platelet endothelial cell adhesion molecule (PECAM1) and vascular endothelial cadherin (VE-CAD/CDH5).^21^^–^^26^ Cells that undergo EndMT lose their ability to perform normal EC functions, disturbing the endothelium. Additionally, by gaining aberrant expression of ECM and mesenchymal proteins, these cells further disrupt the endothelium, exacerbating endothelial dysfunction and downstream effects.

Hyperglycemia-induced EndMT involves regulation through extracellular stimuli, intracellular signaling, as well as epigenetic modulation of responses.^21^^–^^28^ It is well established that EndMT can be induced by profibrotic stimulation via TGF-β, as well as by inflammatory stimulation via proinflammatory cytokines.^21^^–^^26^ Both of these major triggers of EndMT are present in the endothelium under hyperglycemic stress.^19^^,^^20^^,^^29^^,^^30^ High glucose increases TGF-β expression, triggering canonical and non-canonical TGF-β signaling, resulting in EndMT changes.^22^^–^^24^^,^^31^^,^^32^ High glucose also causes inflammation, leading to increased abundance of proinflammatory cytokines and activation of NF-κB signaling, again resulting in EndMT changes.^19^^,^^20^^,^^29^^,^^33^ The pro-EndMT effects of TGF-β and proinflammatory cytokine signaling are further modulated and propagated by hyperglycemia-induced changes in epigenetic regulation.^22^^–^^24^

Epigenetic regulation describes persistent changes in phenotype without underlying changes in genotype. Mechanisms of epigenetic regulation include DNA methylation, histone modifications, and modulation by non-coding RNAs(ncRNAs).^34^^,^^35^ DNA methylation and histone modifications regulate gene expression at the transcriptional level, whereas ncRNAs modulate gene expression at both transcriptional and post-transcriptional levels.^34^^–^^36^ NcRNAs can be divided into many categories, including long non-coding RNAs (lncRNAs) and microRNAs (miRNAs). lncRNAs are long (>200 nucleotides) molecules that fold into sophisticated 3D structures and bind to proteins, serving as scaffolds, guides, or decoys to promote or repress gene expression.^34^^–^^37^ MiRNAs, on the other hand, are short (∼22 nucleotides) molecules that suppress the expression of target mRNAs via translational inhibition or inducing transcript degradation, typically by binding to the 3ʹ UTR region of the target transcript.^34^^,^^35^^,^^38^ MiRNAs and lncRNAs also have reciprocal influences on one another, lncRNAs can prevent miRNA activity by sponging them, on the other hand, miRNAs can degrade lncRNAs by binding to them.^37^^,^^39^^,^^40^

MiR-9 is a conserved microRNA that is involved in a variety of cancers and other diseases.^41^^–^^44^ MiR-9 has been reported to regulate TGF-β signaling via regulation of the type II TGF-β receptor,^45^^,^^46^ and NF-κB-mediated inflammatory signaling via regulation of the NF-κB subunit 1,^47^^–^^49^ making it a molecule of interest with respect to EndMT. MiR-9 has also been reported to regulate the lncRNA, MALAT1, which we previously found to regulate inflammation in DR,^50^^,^^51^ and which others have reported to regulate EndMT in other disease models.^52^^,^^53^ In a previous microRNA microarray,^54^ data showed that miR-9 abundance is reduced in the retinas of diabetic animals. Taken together, miR-9 appears to be intertwined with several EndMT-related processes, yet the direct role of miR-9 in EndMT in DR has never been investigated.

## Material and Methods

### Cells

Human retinal endothelial cells (HREC; Olaf Pharmaceuticals, Worcester, MA, USA) were cultured with endothelial cell growth basal medium-2 (Lonza Group, Basel, Switzerland) supplemented with microvascular endothelial growth medium-2 (Lonza Group), 10% v/v fetal-bovine serum, and 100 µg/mL penicillin/streptomycin in a humidified hood held at 37°C with 4% CO_2_. Once the cells reached 80% to 90% confluency, the full growth medium was aspirated, replaced with serum-reduced medium, and incubated for 24 hours. After 24 hours of serum starvation, cells were treated to normal (NG, 5 mM) or high (HG, 25 mM) glucose concentrations for 48 hours. L-glucose was used as an osmotic control for the HG treatment.

To manipulate miR-9 activity, HRECs were transfected with mirVana miR-9 mimic (Ambion, Austin, TX, USA) or anti-miR-9 miRNA inhibitor (Ambion). Transfection with scrambled miRNA was used as a control. Transfection occurred before serum starvation and glucose treatments using selected oligonucleotides and lipofectamine 2000 (Invitrogen, Carlsbad, CA, USA) in OPTI-MEM medium (Gibco, Thermo Fisher Scientific, Waltham, MA, USA) as previously described.^22^^,^^23^ After six hours of incubation in the transfection mixture, cells were recovered in full growth medium for 24 hours, after which cells were serum starved and treated to varying levels of glucose as mentioned above. The effects of miR-9 mimic and miR-9 inhibitor transfection on miR-9 levels is shown in Supplementary Figure S1.

### Animals

Animals were held in the university facility and cared for according to the Guiding Principles in the Care and Use of Animals. Experimental protocols were approved by Western University Ethics Committee and Animal Care and Veterinary Services. All experiments adhere to the ARVO Statement for the Use of Animals in Ophthalmic and Vision Research. Necessary sample sizes for animals were calculated based on measurements of ECM production as described previously^55^ and reaffirmed via the “resource equation” method.^56^

Tie-2 promoter driven, endothelial-specific, miR-9 transgenic (M9) mice were created and bred in-house. The transgenic vector was created by inserting a cDNA fragment containing miR-9 into the pSPTg.T2FpAXK (#52) plasmid (kindly provided by Thomas Sato; Addgene plasmid no. 35962), which contains a Tie-2 promoter, enhancer, and a SV40 PolyA signal.^57^ A simple vector map is in Supplementary Fig. S2A. The fragment containing Tie-2-miR-9 was excised using the Sall restriction enzyme (Thermo Fisher Scientific) and injected into mouse blastocysts of a C57BL/6(B6) background. Blastocysts were then transferred into pseudopregnant female mice as described.^22^^,^^23^ Six founders were produced, and three pairs were used. One pair of founders was used for downstream studies. Founders for downstream study were chosen for robust miR-9 expression and lack of behavioral or phenotypical changes compared with wild-type (WT) B6 mice. The exact location and copies of transgene insertions were not assessed. Transgenic mice were back crossed with B6 mice and the offspring were genotyped via a polymerase chain reaction (PCR)-based method using genomic DNA from tail-tip biopsy as described^22^^,^^23^ (primers: forward: GCCCTGCTGATACCAAGTG; reverse: GTGCGGCTAGAACATCCA). Subsequent generations were also genotyped using the same method (Supplementary Fig. S2B). The M9 mice showed no behavior or phenotypic differences compared to WT mice. Non-transgenic littermates were used as controls. Both male and female mice were used.

Male and female mice were randomly assigned to either the diabetic group or non-diabetic control group. Diabetes was induced at 8 weeks of age via 5 consecutive daily intraperitoneal injections of streptozotocin (50 mg/kg in citrate buffer, pH4.5; controls received only buffer), as described.^22^^–^^24^ The inclusion criteria for diabetic mice was blood glucose measurements of over 20 mmol/L following the final injection.^22^^–^^24^ Age- and sex-matched nondiabetic controls were used. Mice were monitored for body weight, blood glucose and urine ketone and glucose levels on a weekly basis. After two months, the mice were euthanized, and the eyes were harvested. One eye was fixed in 10% neutral-buffered formalin and embedded in paraffin and used for histological analyses. The remainder of the eyes were stored at −80°C for further analyses. All analyses were performed in a masked fashion.

### Mouse Retinal Cell Isolation

Retinal endothelial cells and non-endothelial cells were isolated from WT and M9 mice as described previously.^22^ Briefly, Dynabeads (Invitrogen) were washed in 0.1% BSA-PBS (B-PBS) for 3 times. The beads were then incubated with 20 µL of rat anti-mouse CD31 monoclonal antibody (Invitrogen) at 4°C for three hours. Anti-CD31 antibody-conjugated Dynabeads (CD31-beads) were washed three times using B-PBS and re-suspended in 200 µL B-PBS on ice for later use. The mice were euthanized, and the retinas were isolated and digested with collagenase A for one hour at 37°C. The mixtures were flushed using 5 mL pipet and transferred to 50 mL tube containing cold DMEM with 2% FBS and pelleted by centrifugation. The pellets were re-suspended in the DMEM with 2% FBS solution, passed through a 100 µm cell strainer, and pelleted again. The cells were further incubated with CD31-beads for one hour at 4°C and washed 5 times using B-PBS. Cells bound to CD31-beads (MRECs) were cultured in 2% gelatin coated 6 well plates (Falcon) in EBM2 with supplemental kit same as above (Lonza) and harvested for analysis. The unbound cells (non-ECs) were collected for analysis.

### miRNA Analysis

Total miRNA was extracted from cells and tissues using the SanPrep Column microRNA Miniprep Kit (Biobasic) following the supplier's instructions. The cDNA was synthesized using specific primers and multiscribe reverse transcriptase (Life Technologies, Carlsbad, CA, USA). TaqMan miR-9 assay (Ambion) was used in real time quantitative PCR (RT-qPCR), in accordance with the manufacturer's instructions, to quantify miR-9 levels. MiR-9 abundance was normalized to that of U6 small nuclear RNA (snRNA) in order to account for variation between samples.^22^^,^^54^

### mRNA and lncRNA Analysis

Total RNA was extracted from cells and tissue samples using TRIzol reagent (Invitrogen) as described.^22^^–^^24^ RNA concentrations were measured using the SpectraMax QuickDrop Spectrophotometer (Molecular Devices, San Jose, CA, USA). Total RNA 2 µg was used to synthesize cDNA using the High Capacity cDNA Reverse Transcription kit (Applied Biosystems, Foster City, CA, USA) according to the manufacturer's instructions. RT-qPCR was performed using the LightCycler 96 system (Roche, Basel, Switzerland) to assess the expressions of target genes(primer sequences in Table 1). Gene expressions were quantified using the standard curve method, and expressions of all genes of interest were normalized to that of β-actin (*ACTB/Actb*).^22^^–^^24^

**Table 1.** Primer Sequences Used for RT-qPCR

Gene	Species Specificity	Sequence (5ʹ–3ʹ)
*ACTB/Actb*	Human/mouse	CCTCTATGCCAACACAGTGC
		CATCGTACTCCTGCTTGCTG
*PECAM1*	Human	AGACAACCCCACTGAAGACGTCG
		CCTCTCCAGACTCCACCACCTTAC
*CDH5*	Human	CTACCAGCCCAAAGTGTGTG
		GTGTTATCGTGATTATCCGTGA
*S100A4*	Human	CAACAGGGACAACGAGG
		CTGGGCTGCTTATCTGGG
*TAGLN*	Human	GGCAGGCCCCAGTAAAGAAG
		TGCCAGCCCACCCAGATT
*Pecam1*	Mouse	ACCGGGTGCTGTTCTATAAGG
		CACCTTGGGCTTGGATACGC
*Cdh5*	Mouse	GTCCACCTTCCACAAATACTC
		CTCCCGATTAAACTGCCCAT
*S100a4*	Mouse	GTCCACCTTCCACAAATACTC
		AAGTTGCTCATCACCTTCTGG
*Acta2*	Mouse	CTACTGCCGAGCGTGAGATTGT
		GTTTCGTGGATGCCCGCTGACT

### Protein Analysis

Western blot was used to analyze protein expression from in vitro samples. Briefly, total protein was extracted from samples using RIPA buffer (Millipore, Burlington, MA, USA) containing protease inhibitor (Roche). Protein concentration was assessed using Pierce bicinchoninic acid assay kit (Thermo Fisher Scientific). Thirty micrograms of protein was resolved via SDS-PAGE and transferred to a polyvinylidene difluoride membrane (Bio-Rad Life Science, Hercules, CA, USA). The polyvinylidene difluoride membrane was blocked and incubated with primary antibodies overnight at 4°C, then with secondary antibody for 1 hour at room temperature(antibody information and dilutions in Table 2). Blots were visualized using Clarity Western ECL Substrate kit (Bio-Rad Life Science) and ChemiDoc MP Imaging System (Bio-Rad Life Science). Quantification was done using Image Lab software(Bio-Rad).

**Table 2.** Antibodies for Western Blot

Antibody	Dilution
Rabbit antibody against β-actin (Abcam, ab8227)	1:1000
Rabbit antibody against PECAM1 (Abcam, ab281583)	1:1000
Mouse antibody against S100A4 (Proteintech, 66489-1-Ig)	1:1000
Goat anti-rabbit IgG-HRP (Santa Cruz, SC-2004)	1:5000
Goat anti-mouse IgG-HRP (Invitrogen, A28177)	1:5000

ELISA was used to quantify protein expression from in vivo samples. Total protein was isolated using RIPA buffer (Millipore). Total protein concentration was assessed using bicinchoninic acid assay (Thermo Fisher Scientific). Pecam1 (ab204527; Abcam, Cambridge, MA, USA) and Acta2 (NBP2-66429; Novus Biologicals, Littleton, CO, USA) ELISA kits were used according to the suppliers’ instructions. Optical densities were measured at 450 nm using the ChroMate Microplate Reader (Awareness Technology, Inc., Palm City, FL, USA).

### Immunofluorescence

Formalin-fixed paraffin embedded tissue sections (5 µm) were blocked and stained with rabbit anti-mouse CD31 (1:200, ab28364; Abcam) and mouse anti-mouse α-SMA (1:400, 14-9760-82; Invitrogen) antibodies overnight. Mouse-on-mouse IgG Blocking Solution (Invitrogen) was used to reduce non-specific binding. The sections were washed and incubated with secondary antibodies (Alexa Fluor 555 goat anti-mouse and Alexa Fluor 488 goat anti-rabbit, 1:200; Invitrogen) for one hour. Fluorescence was examined on a fluorescent microscope (Olympus BX51; Olympus, Tokyo, Japan). Images were taken and processed using the Infinity 3 camera (Lumenera Corporation, Ottawa, Canada) and its associated software.

### Permeability Assay

Tissue sections (5 µm) from formalin-fixed paraffin embedded blocks were immunohistochemically stained with an anti-IgG rabbit antibody (MP Biomedicals, Solon, OH, USA) overnight at 1:1000 dilution. The sections were then incubated with HRP-conjugated secondary antibody (anti-Rabbit IgG Peroxidase Polymer Detection Kit; MJS Biolynx, Brockville, Canada), then with diaminobenzidine (DAB Peroxidase Substrate; MJS Biolynx) for visualization. The slides were counterstained with hematoxylin and examined under a microscope. The strong presence of IgG in the retina was indicative of leakage in retinal capillaries, which are not permeable to IgG in healthy mice. The extent of IgG staining was arbitrarily scored (0, +, ++, +++) in a masked fashion.

### Statistical Analysis

Statistical analyses were done using the open-source software JASP. Statistical significances between multiple groups were determined using one-way ANOVA followed by Tukey's honest significance test for pair-wise comparisons. The threshold of significance (α) was set at *P* ≤ 0.05.

## Results

### High Glucose Promotes EndMT and Represses miR-9 in HRECs

**Figure 1.**
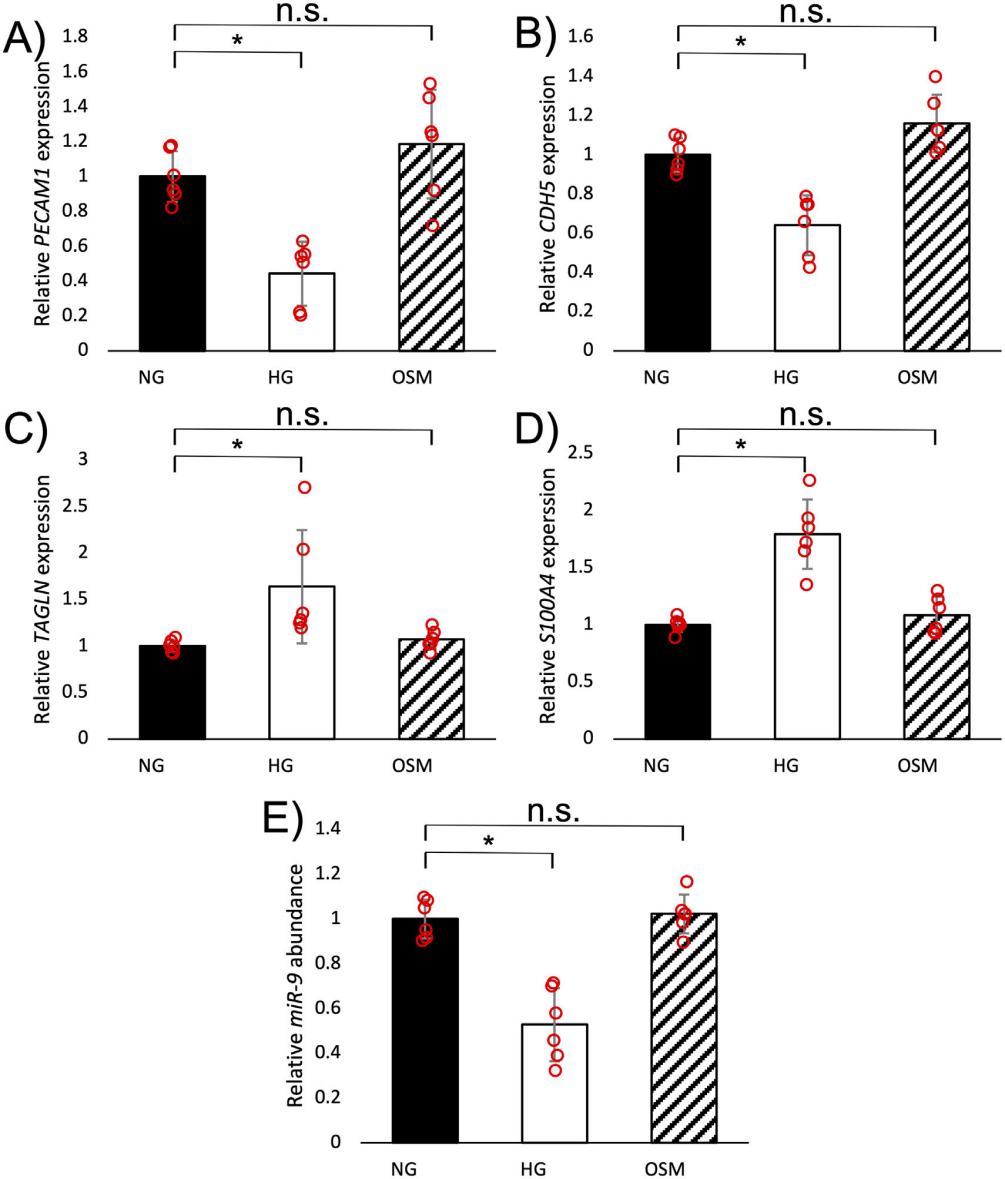
High glucose promoted EndMT and suppressed miR-9 in vitro. Exposure to HG (25 mM for 48 hours) reduced the expressions of endothelial markers (**A**) *PECAM1* and (**B**) *CDH5* and increased the expressions of mesenchymal markers (**C**) *TAGLN* and (**D**) *S100A4* in HRECs. HG also reduced expression of (**E**) miR-9. Exposure to high L-glucose (OSM, 25 mM for 48 hours) produced no significance compared to cells grown in NG (5 mM for 48 hours) (n = 6/group; data presented as ratio to β-actin mRNA or U6 snRNA and normalized to NG; *P < 0.05, n.s., not significant).

To ensure optimal results, we first tested the optimal treatment parameters using various concentrations of glucose and for various time periods(not shown) and confirmed 25 mM glucose for 48 hours was optimal for demonstrating EndMT changes. We found via qPCR that high concentrations of glucose drove EndMT changes in gene expression, significantly increasing the expression of mesenchymal markers *TAGLN* and *S100A4* and decreasing the expression of endothelial markers *PECAM1* and *CDH5* (Figs. 1A–D). At the same time, we confirmed that high glucose concentrations significantly suppressed miR-9 (Fig. 1E). Treatment with 25 mM L-glucose further showed that EndMT is consequence of high glucose specifically and not simply high osmolarity (Fig. 1).

### MiR-9 Mimics Prevent Hyperglycemia-Induced EndMT in HRECs

**Figure 2.**
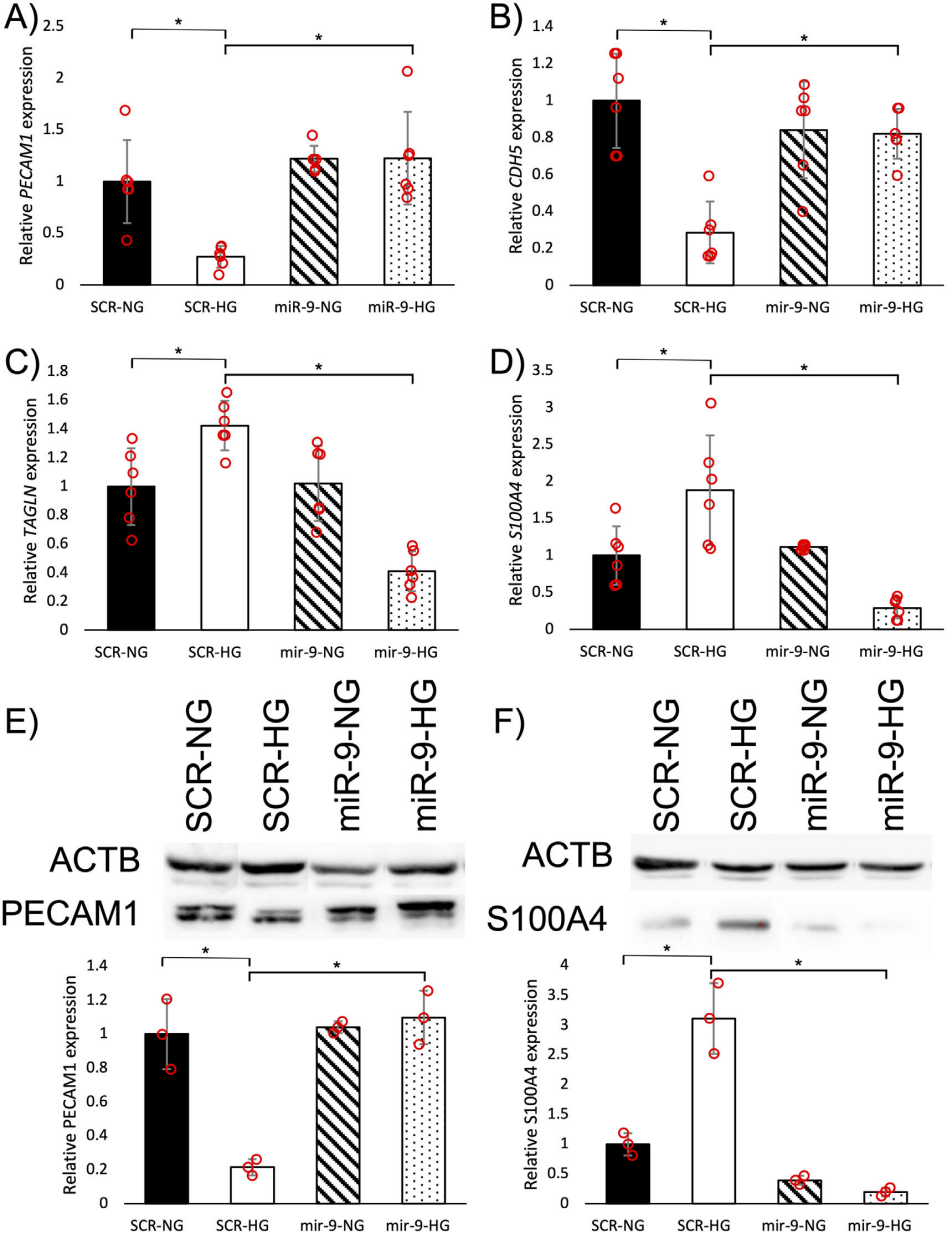
MiR-9 mimics prevented high glucose-induced EndMT changes in vitro. HG (25 mM for 48 hours) significantly reduced mRNA expressions of endothelial markers (**A**) *PECAM1* and (**B**) *CDH5* and increased mRNA expressions of mesenchymal markers (**C**) *TAGLN* and (**D**) *S100A4* in cells transfected with scrambled RNA (SCR). Protein expressions reflect a similar trend, expression of (**E**) PECAM1 was decreased and expression of (**F**) S100A4 was increased in SCR-HG compared with SCR cells cultured under NG (5 mM for 48 hours) conditions. MiR-9 mimic transfection (miR-9) prevented high glucose-induced EndMT changes at both mRNA and protein levels (n = 6/group for mRNA and n = 3/group for protein; data presented as ratio to β-actin mRNA/protein and normalized to NG; *P < 0.05).

Having shown that high glucose both promotes EndMT and suppresses miR-9, we wanted to establish a link between the two occurrences. We transfected HRECs with miR-9 mimics to induce high miR-9 activity. In cells transfected with scrambled RNA, high glucose caused significant downregulation of endothelial markers and upregulation of mesenchymal markers at both RNA and protein levels (Fig. 2). By maintaining high miR-9 activity in high glucose conditions, we prevented high glucose-induced changes. High glucose did not significantly decrease the expressions of endothelial markers in miR-9-transfected cells, nor did it increase the expression of mesenchymal markers (Fig. 2).

### MiR-9 Overexpression Prevents Hyperglycemia-Induced EndMT in the Retinas of Diabetic Mice

**Figure 3.**
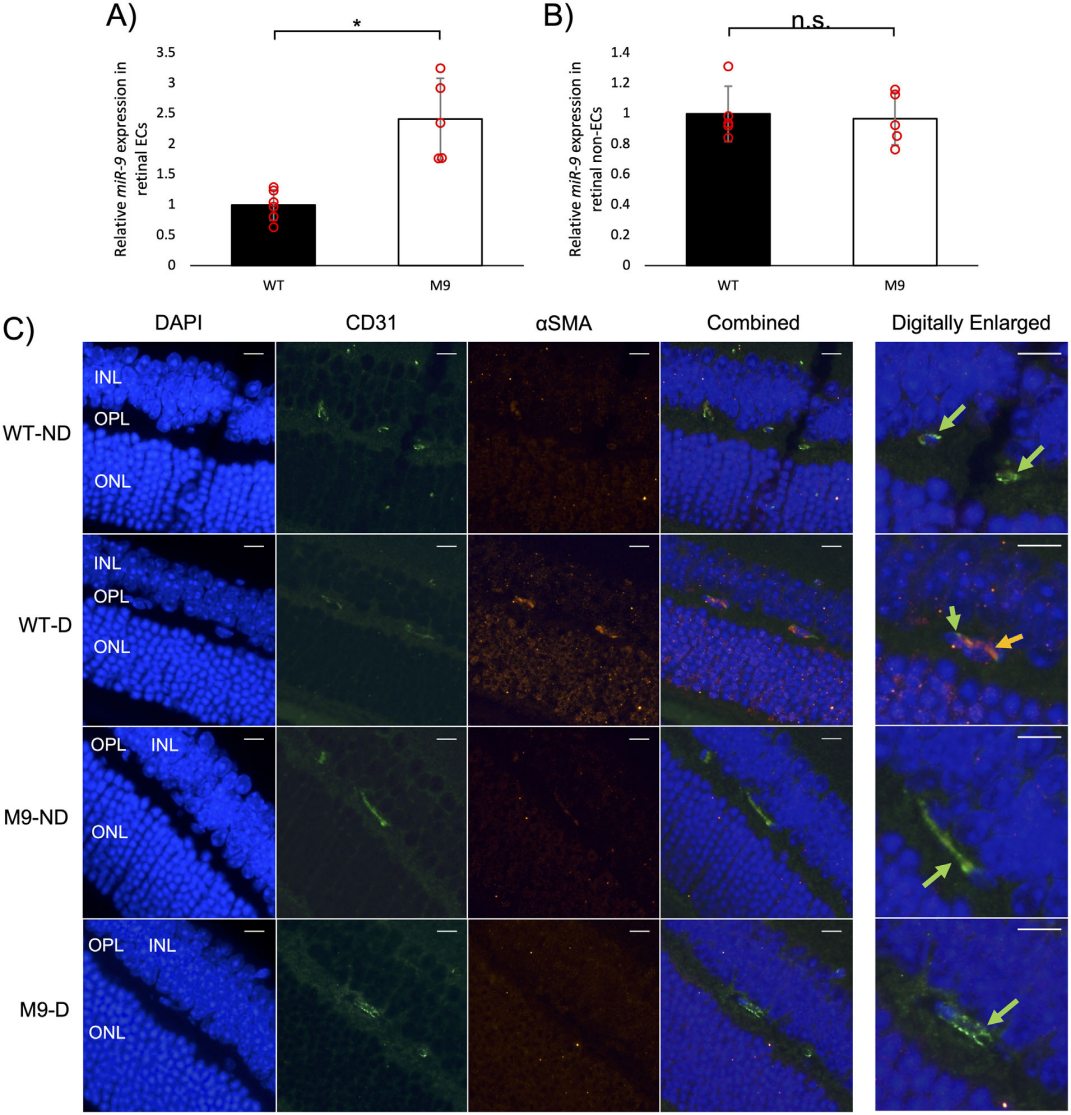
MiR-9 transgenic mice showed endothelial specific miR-9 overexpression and inhibition of EndMT. MiR-9 expression in (**A**) retinal ECs are considerably higher in M9 mice compared with WT mice, whereas miR-9 expression in (**B**) retinal non-ECs showed no significant difference between M9 and WT. (**C**) The endothelial marker CD31 (*green arrows*) showed strong fluorescence in the capillaries of all nondiabetic retinas, as well as in the capillaries of diabetic M9 retinas, and weak fluorescence in the WT diabetic retinas. The mesenchymal marker α-SMA (*orange arrow*) showed strong fluorescence throughout the retinas of diabetic WT mice, and little to no fluorescence in the capillaries of the other groups. There was considerable overlap between red and green fluorescence in diabetic WT retinas. (n = 6/group for miR-9 analysis and n = 4/group for imaging; miR-9 expression presented as ratio to U6 snRNA and normalized to WT; *P < 0.05, n.s., not significant; fluorescence images were uniformly adjusted to reduce background noise; INL, inner nuclear layer; OPL, outer plexiform layer; ONL, outer nuclear layer; *white bar:* 10 µm].

After establishing a link between miR-9 abundance and EndMT in vitro, we wanted to replicate the findings in vivo. We created and validated the transgenic M9 mouse model, which showed endothelial-specific overexpression of miR-9 compared to WT mice (Figs. 3A, 3B). After validating the model, we assessed the effects of miR-9 overexpression on EndMT in DR. Diabetic mice, both WT and M9, had reduced body weight and increased urine volume compared with nondiabetic controls (not shown). We first used immunofluorescent staining for qualitative assessment of EndMT (Fig. 3C). Nondiabetic WT and M9 mice showed intense fluorescence for CD31 (green), and minimal fluorescence for α-SMA (red). Diabetic M9 mice showed similar patterns to nondiabetic WT and M9 mice, whereas diabetic WT mice showed weaker green fluorescence and stronger red fluorescence colocalized to the retinal capillaries, indicating EndMT.

**Figure 4.**
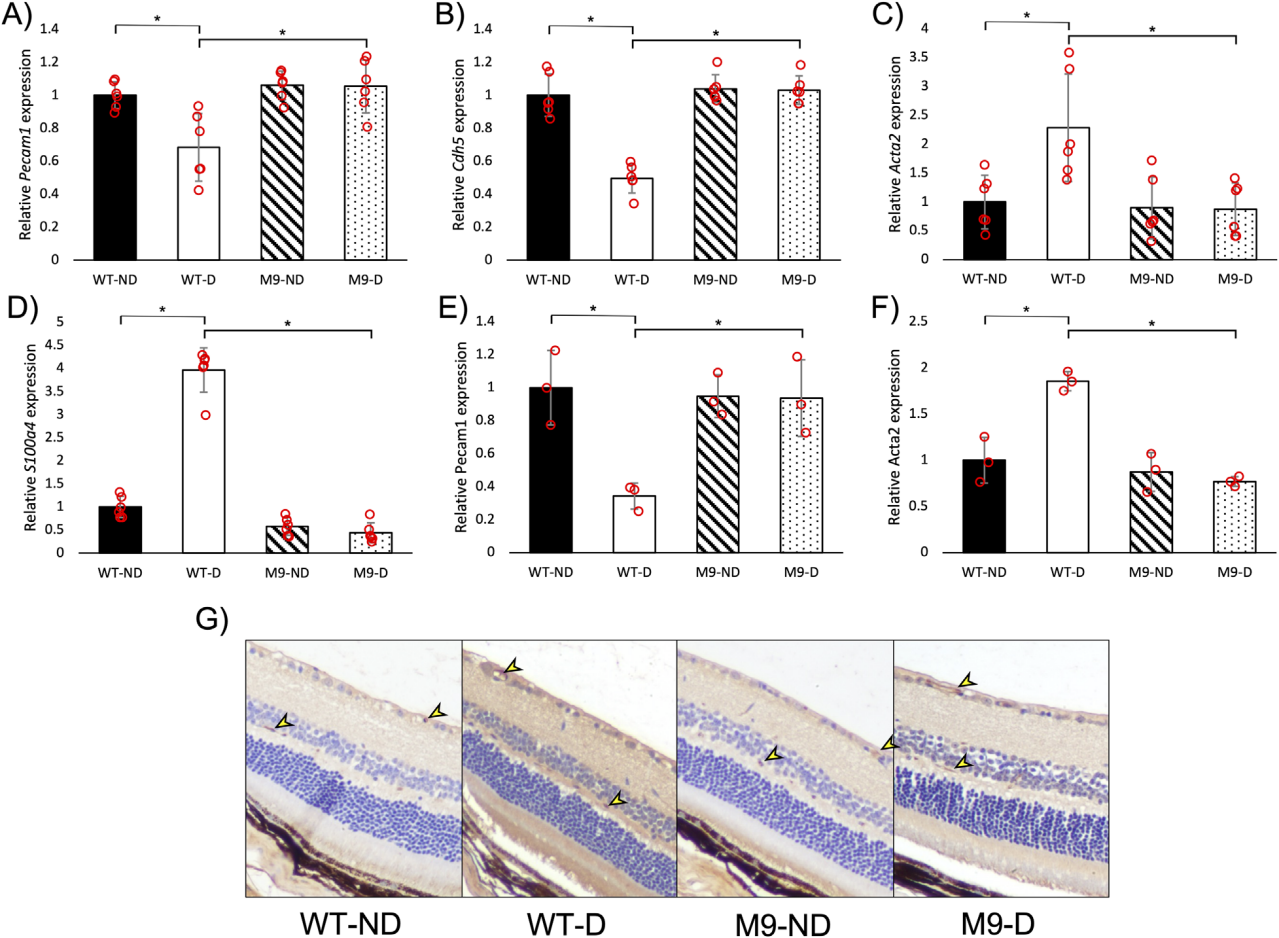
MiR-9 prevented diabetes-induced EndMT and diabetes-induced vascular leakage in mouse retinas. Streptozotocin-induced diabetes significantly reduced retinal mRNA expressions of endothelial markers (**A**) *Pecam1* and (**B**) *Cdh5*, and increased expressions of mesenchymal markers (**C**) *Acta2* and (**D**) *S100a4* in male WT mice. Such changes were prevented in diabetic M9 mice. Protein expressions of (**E**) Pecam1 and (**F**) Acta2 also followed the same pattern. (**G**) IgG staining was seen within the retinal capillaries of all mice (*arrow*). Diabetes caused leakage of IgG into the retinal layers of WT diabetic mice, resulting in intense (+++) staining throughout the retina when compared with the nondiabetic mice (+). Diabetic M9 mice showed minimal leakage of IgG into the retina and had staining intensity comparable to non-diabetic M9 mice (+). ND, nondiabetic; D, diabetic; M9, miR-9 transgenic (n = 6/group for mRNA, n = 3/group for protein, and n = 3/group for immunohistochemistry; molecular data normalized to WT-ND, mRNA data presented as ratio to β-actin mRNA, and protein data presented as ratio to total protein; *P < 0.05).

After immunofluorescent assessment, we conducted qualitative analyses using qPCR and ELISA. On the molecular level, there were no differences in the expressions of endothelial and mesenchymal markers between non-diabetic WT and M9 mice (Figs. 4A–F). Diabetes induced EndMT in WT mice, expressions of endothelial markers were significantly reduced, and expressions of mesenchymal markers were significantly increased on both RNA and protein levels (Figs. 4A–F). Diabetes-induced changes were prevented in M9 mice, expressions of endothelial and mesenchymal markers were not different from non-diabetic M9 mice (Figs. 4A–F). The anti-EndMT effects of miR-9 were consistent between male and female mice (Supplementary Figs. S3A–D).

### Inhibition of EndMT by miR-9 Prevents Vascular Leakage in the Retinas of Diabetic Mice

In order to understand the functional benefits of high miR-9 preventing EndMT in the retina, we used immunohistochemical staining against IgG to assess vascular leakage. Non-diabetic WT and M9 retinas showed similar prevalence of intravascular IgG staining and no leakage (Fig. 4G). Diabetic WT mice showed darker staining throughout the retina, indicating leakage of IgG out of the capillaries into the retina (Fig. 4G). Such vascular leakage was prevented in diabetic M9 mice (Fig. 4G). Male and female mice showed similar patterns in diabetes-induced retinal vascular leakage, and vascular leakage was similarly prevented by miR-9 overexpression in both sexes (Supplementary Fig. S3E).

### MiR-9 Regulates EndMT by Regulating TGF-β and Inflammatory Responses

**Figure 5.**
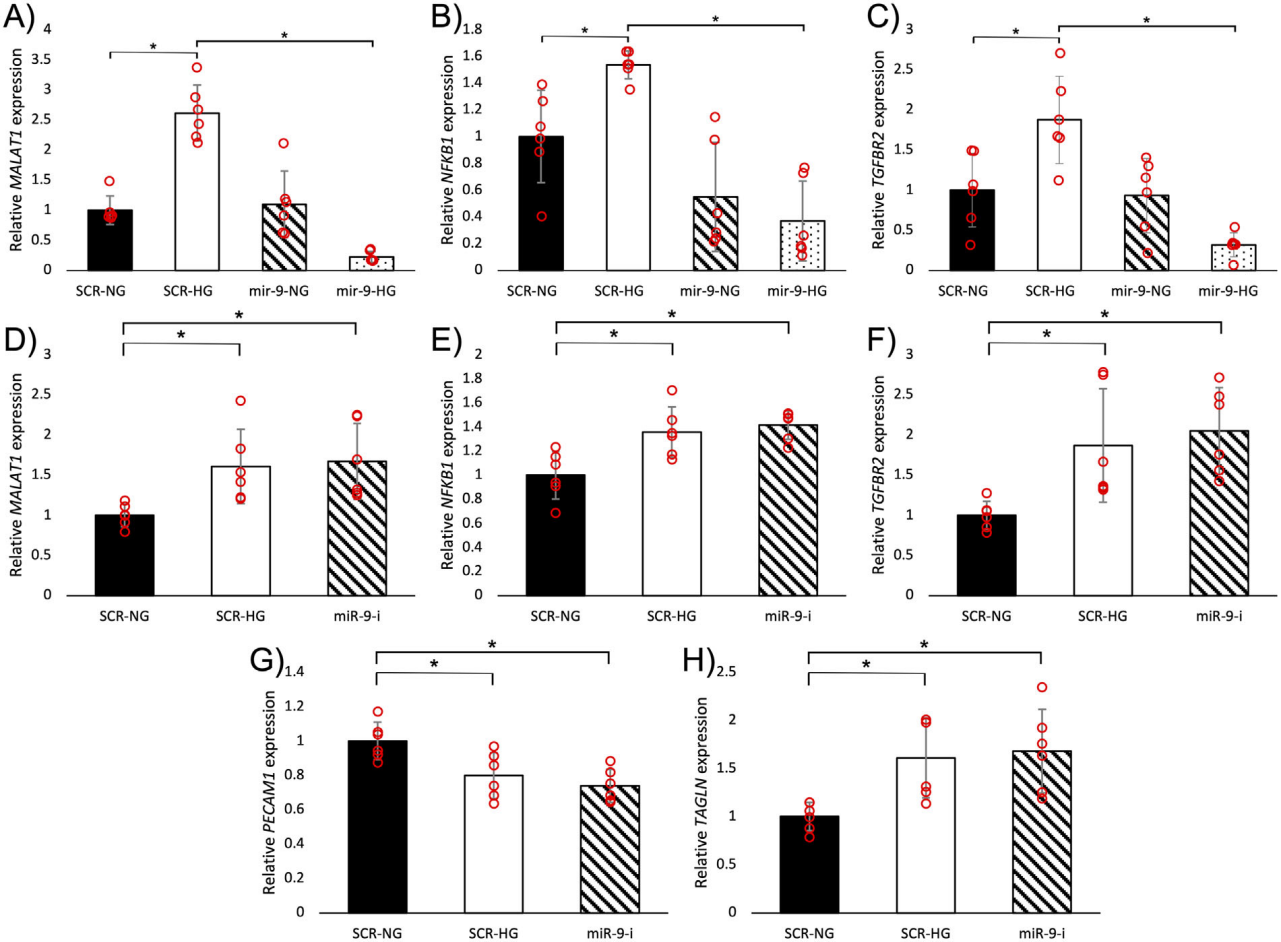
MiR-9 regulates TGF-β and inflammatory signaling to regulate EndMT in HRECs. High glucose (HG, 25mM for 48 hours) induced upregulation of (**A**) *MALAT1,* (**B**) *NFKB1* and (**C**) *TGFBR2,* these changes were prevented by miR-9 mimic transfection. MiR-9 inhibition caused upregulation of (**D**) *MALAT1,* (**E**) *NFKB1,* and (**F**) *TGFBR2* under normal glucose (NG, 5 mM for 48 hours) conditions. Inhibition of miR-9 (miR-9-i) under normal glucose conditions produced a glucose-like effect and induced EndMT-like changes such as reduced expression of (**G**) PECAM1 and increased expression of (**H**) TAGLN (n = 6/group; data presented as ratio to β-actin mRNA and normalized to SCR NG; *P < 0.05).

To gain insight into the mechanisms by which miR-9 regulates EndMT, we took a closer look at some pro-EndMT pathways in vitro. miR-9 mimics were used to determine which aspects of pro-EndMT signaling were regulated by miR-9. HRECs were transfected with miR-9 mimics and treated to normal or high glucose. High glucose caused increased expression of TGF-β receptor II (*TGFBR2*), NF-κB subunit 1 (*NFKB1*), and *MALAT1* in control but not in miR-9-transfected cells (Figs. 5A–C).

Anti-miR-9 microRNA inhibitors were used to determine if miR-9 is a driver or an intermediate regulator of EndMT. MiR-9 inhibition produced high glucose-like effects in HRECs cultured under normal glucose concentrations. Cells transfected with miR-9 inhibitor showed increased expressions of *TGFBR2, NFKB1,* and *MALAT1* (Figs. 5D–F); these changes corresponded with changes in select EndMT markers. MiR-9 inhibition resulted in reduced *PECAM1* expression and increased *TAGLN* expression at a similar level to high glucose treatment (Figs. 5G, %H).

## Discussion

Glucose-induced endothelial changes are among the earliest occurrences in DR and are important in paving the way for further dysfunction. In this study, we investigated the importance of miR-9 in mediating glucose-induced EndMT in early DR. We showed that high glucose promoted endothelial dysfunction in the form of EndMT through the inhibition of miR-9. We further demonstrated that maintaining high miR-9 activity in the endothelium under hyperglycemic conditions prevents EndMT and associated vascular leakage. Finally, we showed that miR-9 regulates multiple pathways leading to EndMT, and that miR-9 inhibition is sufficient to drive EndMT.

Increased glucose metabolism in ECs leads to increased oxidative stress and the aberrant activation of various pathways, resulting in changes in gene regulation and creating an environment that is conducive to EndMT.^19^^,^^20^^,^^23^^,^^24^^,^^29^ Reactive oxygen species generated in the mitochondria and advanced glycation end-products derived from non-enzymatic glycation of proteins trigger inflammation.^19^^,^^20^^,^^58^ Additionally, the activation of the protein kinase C pathway by diacylglycerol derived from the glycolytic intermediate dihydroxyacetone phosphate, leads to the upregulation of NF-κB and TGF-β, among other things.^19^^,^^20^ The existence of glucose-induced EndMT has been known for over a decade, although the mechanisms through which it occurs and its importance in DR has yet to be fully elucidated.

We have previously shown that lncRNA H19 and miR-200b are negative regulators of glucose-induced EndMT in the retina.^23^^,^^24^ Both H19 and miR-200b inhibit glucose-induced EndMT by inhibiting TGF-β signaling; H19 inhibits non-canonical TGF-β signaling through the MAPK-ERK pathway, while miR-200b interferes with canonical TGF-β signaling through SMAD2.^23^^,^^24^ miR-9 is a well-conserved miRNA that is involved in the pathogenesis of several cancers.^43^^,^^44^^,^^48^^,^^49^^,^^59^ It became a molecule of interest during our investigation of miR-200b. Various studies have reported the role of miR-9 in regulating EndMT-adjacent processes such as proinflammatory signaling and TGF-β signaling, inhibiting,^45^^–^^47^^,^^59^^–^^61^ but few have investigated miR-9 in the context of diabetic complications. MiR-9 has been reported to directly inhibit NFKB1, TGFBR2, and MALAT1 through complementarity-based silencing.^45^^–^^47^^,^^59^^–^^61^ NFKB1 encodes the DNA binding subunit of the canonical NF-κB complex. Increased NF-κB activity is seen in the vasculature during hyperglycemia and is associated with the progression of EndMT.^62^^–^^64^ TGFBR2 encodes the type II TGF-β receptor, which with its counterpart, type I TGF-β receptor, propagates TGF-β signaling. We have previously demonstrated that TGF-β signaling is a key driver of EndMT in the retina,^23^^,^^24^ and others have found that partial deletion of TGFBR2 alleviated TGF-β-induced EndMT in other systems.^65^ MALAT1 is a highly conserved lncRNA found in high abundance in the nucleus.^50^^,^^51^^,^^66^ We have previously shown that MALAT1 is an epigenetic regulator of inflammation in DR,^50^ and others have reported that MALAT1 modulates EndMT through a variety of means.^52^^,^^53^

MiR-9 is just one of the multitude of transcripts that are repressed under high glucose conditions, but unlike most of the other differentially expressed genes, miR-9 is a key factor linking increased glucose to EndMT and its downstream effects. Through our experiments, we found that miR-9 exerts its effect on EndMT at a relatively early stage, acting like a gatekeeper. Under normal conditions, higher levels of miR-9 modulate TGF-β and proinflammatory signaling. Glucose-induced suppression of miR-9 lifts the inhibition of TGF-β and proinflammatory signaling, allowing for pro-EndMT signaling to occur, resulting in EndMT. Maintaining high levels of miR-9 under high glucose conditions shuts the door on pro-EndMT signaling and prevents EndMT from occurring. On the other hand, inhibition of miR-9 promotes inflammation by increasing MALAT1 expression and increasing the sensitivity of ECs to TGF-β and NF-κB signaling, resulting in EndMT changes even under normal glucose conditions. In this study we delved into the latter half of the glucose-miR-9-EndMT axis, focusing on the relation between miR-9 and EndMT. Further investigation into the mechanistic link between high glucose and miR-9 suppression will be required to fully elucidate the glucose-miR-9-endMT pathway in the retina. Our laboratory has recently demonstrated a link between glucose-induced upregulation of lncRNA ZFAS1 and the suppression of miR-9 in cardiac fibrosis,^67^ but this relation needs to be validated in DR in future experiments.

When ECs undergo EndMT and lose their endothelial characteristics, they lose their ability to support the various functions of normal ECs. In the retinal vasculature, ECs have the critical role of forming and maintaining the inner component of the blood-retina barrier (BRB).^68^ The BRB insulates the environment of retina from normal circulation, limiting the passage of ions, drugs, and other molecules in the eye, in order to maintain strict homeostatic control in the retina.^68^^,^^69^ Furthermore the BRB blocks inflammatory mediators and immune cells, preventing potentially harmful intraocular inflammation and helping to enforce a so-called immune privileged status of the retina.^68^^,^^69^ The loss of EC barrier functions in the retina via EndMT, combined with other factors, compromises the BRB and leads to loss of immune privilege and increased vascular leakage.^68^^,^^69^ Increased vascular permeability is one of the early symptoms of DR and has been associated with increased risk of diabetic macular edema and vision loss.^68^^,^^70^ Loss of immune privilege allows for immunological agents to flow across the BRB. Inflammation in the retina can cause damage to neuronal tissues leading to visual impairment, and can further disrupt the retinal vasculature, contributing to vascular occlusion and neovascularization further down the line.^7^^,^^9^^,^^11^^,^^71^

The importance of EndMT in various aspects of DR is not fully understood. Research has shown that pathologic EndMT does not occur in all ECs of a vessel, nor is the proportion of ECs undergoing EndMT consistent across organs.^72^^–^^74^ It is difficult therefore to translate in vitro findings into biologically relevant outcomes. The only way to confirm the benefits of miR-9-mediated EndMT prevention is by using experimental animal models. Using our M9 mice, we found that miR-9 overexpression in diabetic mice prevented EndMT and DR-associated vascular leakage. This shows that EndMT contributes to the breakdown of the BRB and that miR-9 overexpression helps maintain BRB integrity, despite not knowing the exact proportion of ECs that underwent EndMT. Although in vivo experiments are not without their own potential limitations. We recognize that in molecular analyses, EndMT markers from the animals were analyzed in whole retinas rather than in isolated ECs, which might have diluted the perceived magnitude of the change in these markers. However, combined with immunofluorescence analysis and in vitro experiments, the data support the overall notion of EndMT in DR and the role of miR-9.

The protective effect of miR-9 overexpression against vascular leakage in DR without causing noticeable side effects makes it a good potential candidate for RNA-based therapy in diabetic patients. MiR-9 has been studied in cancer research and has been assessed as a potential biomarker and therapeutic in several cancers.^44^^,^^75^^,^^76^ MiRNA-based therapies can be advantageous because of their ability to regulate a wider network of transcripts, rather than specific genes. Therapeutic miRNAs or miRNA mimics can be delivered via lipid nanoparticles or can be conjugated to carbohydrates and taken up by target cells.^77^^,^^78^ MiR-9 therapy, alone or combined with existing therapies such as anti-VEGF agents, may potentially provide benefits to patients with diabetic patients by inhibiting vascular leakage. This may be particularly beneficial for patients with diabetic macular edema, a complication of DR which often does not respond favorably to anti-VEGF therapy.^79^ Given the scope and design parameters of our current study, two-month diabetic model, we are limited to looking at the early developments of DR. Diabetic mice suffer from ketosis beyond the two-month mark and will require interventions that may introduce confounding variables. In this study, we aimed to conduct experiments without such confounding factors as a foundation for future experiments. Further investigations evaluating long-term changes such as acellular capillaries and vascular cell apoptosis, as well as the effect of miR-9 on diabetic macular edema will be required to fully appreciate the benefits of miR-9 and to assess its possibility for use as a therapeutic miRNA.

In summary, we have shown that high glucose-induced EndMT in retinal ECs is concurrent with suppression of miR-9, and that miR-9 is a key regulator of glucose-induced EndMT. We have further shown that miR-9 inhibits EndMT by suppressing members of EndMT-inducing signalling pathways such as *TGFBR2*, *NFKB1*, and *MALAT1*. Finally, we showed that preventing EndMT by maintaining miR-9 abundance can prevent glucose-induced vascular leakage in the retina. Although we do not yet have a complete mechanistic understanding of the regulation of glucose-induced EndMT, our findings highlight miR-9 as an important player and as a potential molecule for targeted RNA-based gene therapy approaches in DR.

## Supplementary Material

Supplement 1

Supplement 2

Supplement 3
