# Supplementary material for: MicroRNA 9 Is a Regulator of Endothelial to Mesenchymal Transition in Diabetic Retinopathy
Source: Invest Ophthalmol Vis Sci. 2023 Jun 6;64(7):13. doi: 10.1167/iovs.64.7.13 (PMC10249683; doi:10.1167/iovs.64.7.13)
Supplement: Supplement 1 [file iovs-64-7-13_s001.pdf]

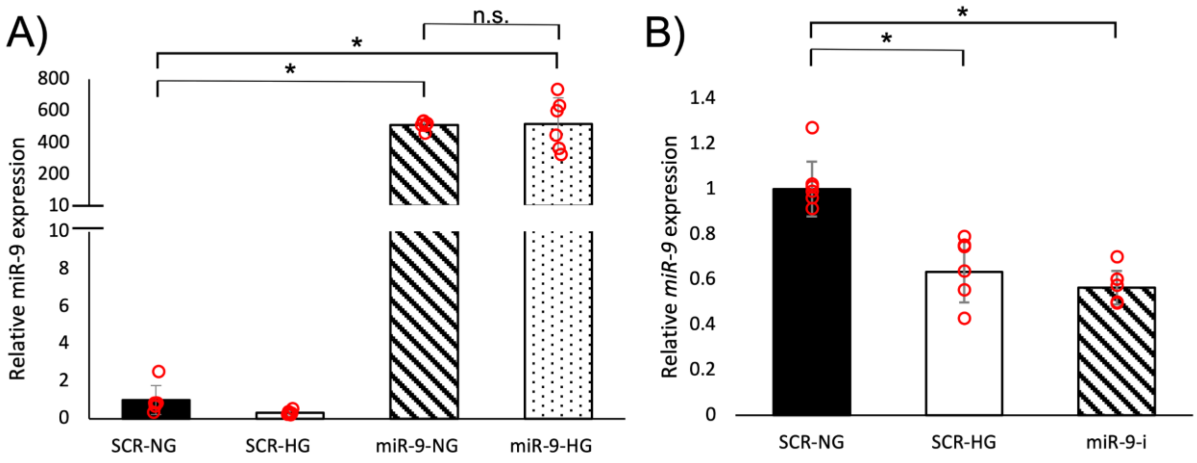

Supplemental figure 1. Quantitation of miR-9 following transfection with mimics or inhibitors. A) Transfection with miR-9 mimic produced a 500-fold increase in miR-9 abundance relative to cells transfected with scrambled RNA (SCR). High glucose (25mM for 48 hours) caused a 50% decrease in miR-9 abundance in SCR-transfected cells, in line with what is observed in cells without transfection, and produced no difference in mimic-transfected cells. B) Transfection with miR-9 inhibitors (miR-9-i) caused a 50% decrease in miR-9 abundance, compared to SCR-transfected cells cultured under normal glucose conditions (5mM for 48 hours), similar to the effect produced by high glucose. [n=6/group; data presented as ratio U6 snRNA and normalized to SCR-NG; * = p<0.05, n.s.= not significant]
