# Supplementary material for: MicroRNA 9 Is a Regulator of Endothelial to Mesenchymal Transition in Diabetic Retinopathy
Source: Invest Ophthalmol Vis Sci. 2023 Jun 6;64(7):13. doi: 10.1167/iovs.64.7.13 (PMC10249683; doi:10.1167/iovs.64.7.13)
Supplement: Supplement 2 [file iovs-64-7-13_s002.pdf]

A)

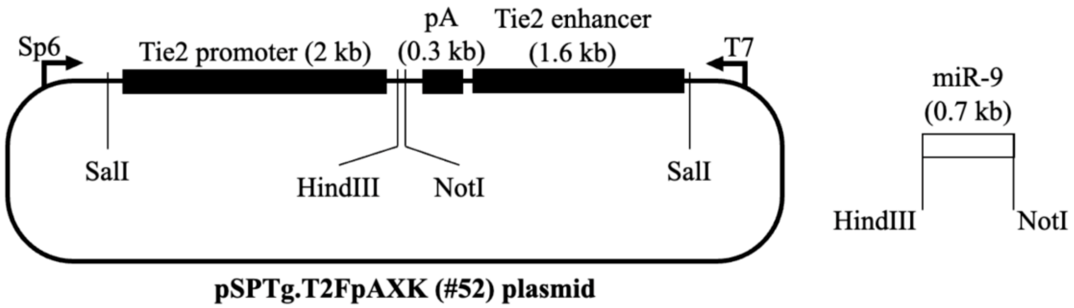

B)

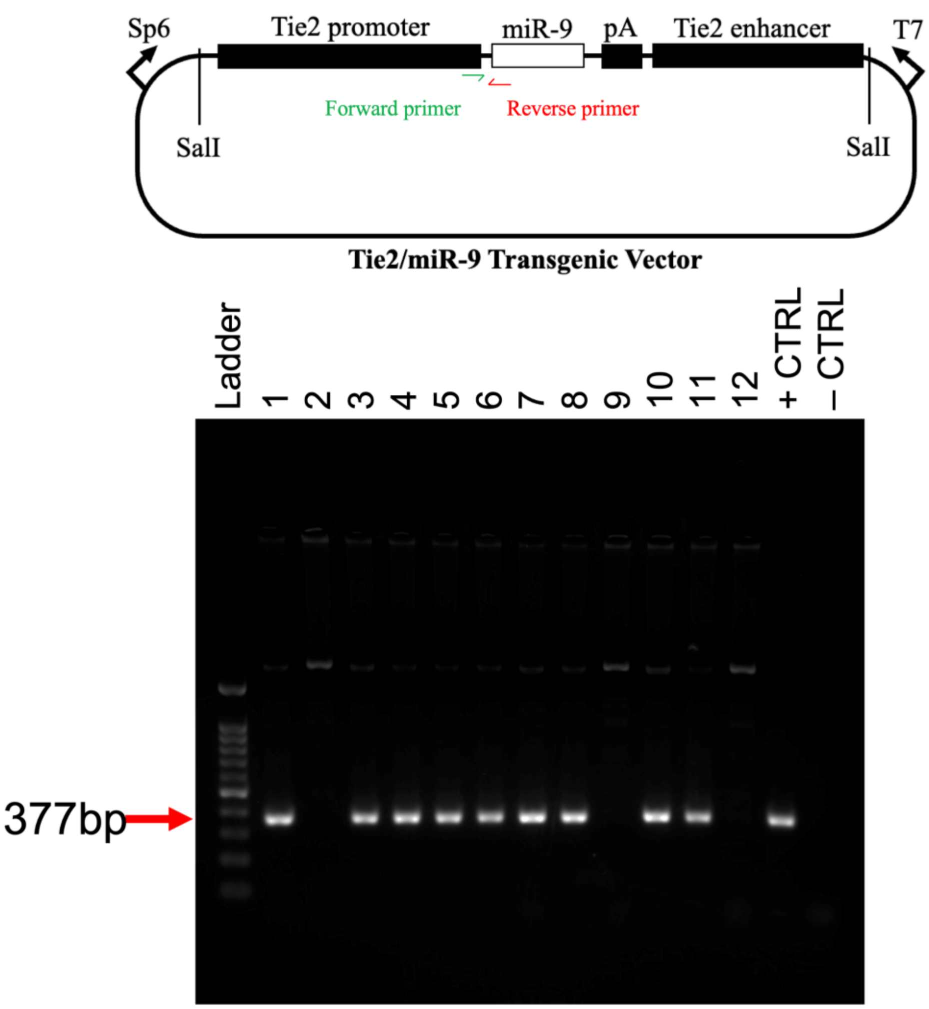

Supplemental figure 2. Transgenic mouse information. A) Vector map of the transfection vector used to generate Tie2- promoter-driven miR-9 transgenic (M9) mice. A 0.7 kb fragment containing miR-9 was inserted into the pSPTg.T2FpAXK (#52) plasmid using HindIII and NotI restriction sites. The final vector contains a Tie2 promoter, miR-9, polyadenylation signal, and a Tie2 enhancer. B) Genotyping for M9 mice. A 377bp fragment within the transgenic segment was amplified via PCR. Animals showing intense bands at 377bp were positive for Tie-2-driven miR-9 overexpression.
