# Supplementary material for: MicroRNA 9 Is a Regulator of Endothelial to Mesenchymal Transition in Diabetic Retinopathy
Source: Invest Ophthalmol Vis Sci. 2023 Jun 6;64(7):13. doi: 10.1167/iovs.64.7.13 (PMC10249683; doi:10.1167/iovs.64.7.13)
Supplement: Supplement 3 [file iovs-64-7-13_s003.pdf]

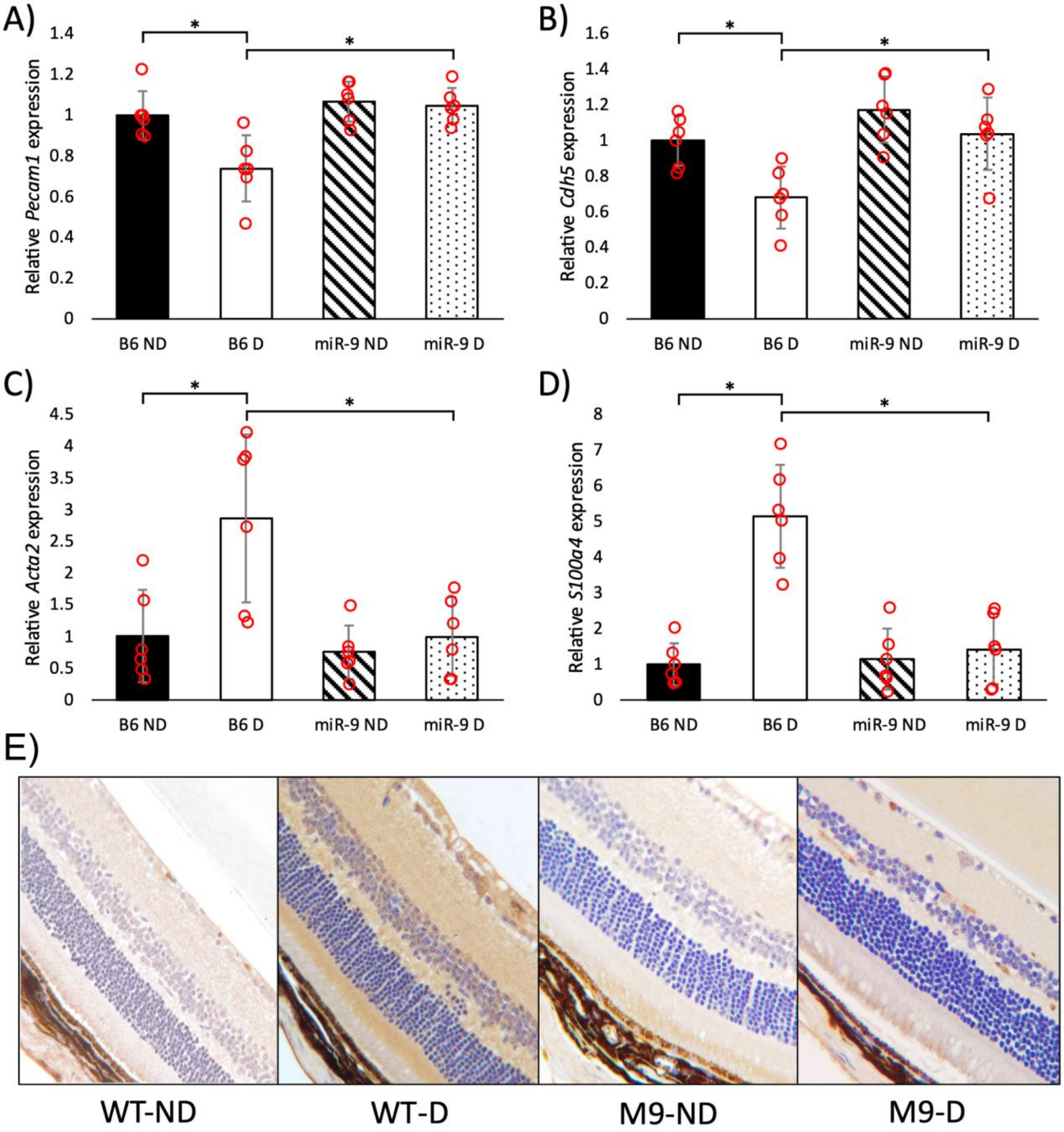

Supplemental figure 3. miR-9 prevented diabetes-induced EndMT and diabetes-induced vascular leakage in the retinas of female mice. STZ-induced diabetes significantly reduced retinal mRNA expressions of endothelial markers A) *Pecam1* and B) *Cdh5*, and increased expressions of mesenchymal markers C) *Acta2* and D) *S100a4* in female WT mice. Such changes were prevented in diabetic M9 mice. E) IgG staining was seen within the retinal capillaries of all mice (arrow). Diabetes caused leakage of IgG into the retinal layers of female WT diabetic mice, resulting in intense (+++) staining throughout the retina when compared with the non-diabetic mice (+). Female diabetic M9 mice showed minimal leakage of IgG into the retina and had staining intensity comparable to non-diabetic M9 mice (+).
[n=6/group for mRNA, n=3/group for protein, and n=3/group for immunohistochemistry; molecular data normalized to WT-ND, mRNA data presented as ratio to β-actin mRNA, and protein data presented as ratio to total protein; * = $p<0.05$.]
